# The Sex and Race Specific Relationship between Anthropometry and Body Fat Composition Determined from Computed Tomography: Evidence from the Multi-Ethnic Study of Atherosclerosis

**DOI:** 10.1371/journal.pone.0139559

**Published:** 2015-10-08

**Authors:** Morgana Mongraw-Chaffin, Sherita Hill Golden, Matthew A. Allison, Jingzhong Ding, Pamela Ouyang, Pamela J. Schreiner, Moyses Szklo, Mark Woodward, Jeffery Hunter Young, Cheryl A. M. Anderson

**Affiliations:** 1 Department of Family Medicine and Public Health, School of Medicine, University of California San Diego, La Jolla, CA, United States of America; 2 Department of Epidemiology, Johns Hopkins Bloomberg School of Public Health, Baltimore, MD, United States of America; 3 Department of Medicine, The Johns Hopkins University School of Medicine, Baltimore, MD, United States of America; 4 Geriatrics and Gerontology, Wake Forest School of Medicine, Winston-Salem, NC, United States of America; 5 Division of Epidemiology and Community Health, University of Minnesota, Minneapolis, MN, United States of America; 6 The George Institute for Global Health, The University of Sydney, Sydney, Australia; 7 The George Institute for Global Health, Nuffield Department of Population Health, University of Oxford, Oxford, United Kingdom; Weill Cornell Medical College in Qatar, QATAR

## Abstract

**Background:**

Few studies have investigated the relationship of anthropometric measurements with computed tomography (CT) body fat composition, and even fewer determined if these relationships differ by sex and race.

**Methods:**

CT scans from 1,851 participants in the population based Multi-Ethnic Study of Atherosclerosis were assessed for visceral and subcutaneous fat areas by semi-automated segmentation of body compartments. Regression models were used to investigate relationships for anthropometry with visceral and subcutaneous fat separately by sex and race/ethnicity.

**Results:**

Participants were 50% female, 41% Caucasian, 13% Asian, 21% African American, and 25% Hispanic. For visceral fat, the positive relationship with weight (p = 0.028), waist circumference (p<0.001), waist to hip ratio (p<0.001), and waist to height ratio (p = 0.05) differed by sex, with a steeper slope for men. That is, across the range of these anthropometric measures the rise in visceral fat is faster for men than for women. Additionally, there were differences by race/ethnicity in the relationship with height (p<0.001), weight (p<0.001), waist circumference (p<0.001), hip circumference (p = 0.006), and waist to hip ratio (p = 0.001) with the Hispanic group having shallower slopes. For subcutaneous fat, interaction by sex was found for all anthropometric indices at p<0.05, but not for race/ethnicity.

**Conclusion:**

The relationship between anthropometry and underlying adiposity differs by sex and race/ethnicity. When anthropometry is used as a proxy for visceral fat in research, sex-specific models should be used.

## Introduction

Since anthropometry is relatively cheap, easy to measure, and non-invasive, traditional measures of adiposity have become standard tools for research and medicine, with body mass index (BMI) being most commonly used.[1] Because it is assumed that they are reasonable approximations of underlying body fat composition and, therefore, valid indicators of the risk associated with varying levels of adiposity, the use of these measurements is ubiquitous. Criticisms about the use of BMI to approximate body fat composition include its imperfect correlation with adiposity in general, and more specifically, its inability to distinguish between visceral and subcutaneous adiposity,[2] with visceral fat playing a larger role in cardiometabolic disease than adipose stored in subcutaneous depots.[2–7] While the debate continues about the best anthropometric measurement to predict cardiovascular risk, there is little research to show how commonly used anthropometric measurements are related to underlying body fat composition. Many of the criticisms of BMI may apply to other anthropometric measures as well. Further, the use of these measures to approximate underlying adiposity without understanding the relationship between them might compromise the evidence for how adiposity relates to health risks. This is particularly likely as the field moves to investigate more factors that have small true effects but population wide influence.

There has also been debate about whether the true relationship between anthropometry and body fat composition differs by sex and race,[8–10] with some data suggesting that anthropometric measures may perform less well for certain groups. If true, this failure to approximate body fat composition with the same accuracy in all groups could limit the ability to explain disparities in cardiovascular disease (CVD) by sex and race. While using separate waist circumference cut-points for men and women has become standard, controversy remains over use of race-specific cut-points for other anthropometric indices. The evidence used to recommend subgroup-specific cut-points for men and women comes primarily from studies investigating risk of health outcomes,[3, 11] but the reasons for such differences in risk at the same level of anthropometry beyond skeletal structure have yet to be fully investigated. Furthermore, very few studies have provided evidence about race and sex differences in the relationships between anthropometric measures and underlying body fat composition.[12–14]

The current study reported the relationships between commonly used anthropometric measures and body fat composition determined by computed tomography (CT) in the Multi-Ethnic Study of Atherosclerosis (MESA) study, and whether they differed by sex and race/ethnicity.

## Methods

### Study Population and Sample Selection

The Multi-Ethnic Study of Atherosclerosis (MESA) is a US community based longitudinal multi-site study with baseline data collection on 6,814 participants free of known cardiovascular diseases and aged 45–84 years in 2000 to 2002.[15] Complete documentation about the MESA study can be found at: http://www.mesa-nhlbi.org/aboutMESA.aspx. The current analysis includes participants from the Abdominal Body Composition, Inflammation and Cardiovascular Disease ancillary study, which included a 30% random sample (N = 1,975) of the MESA cohort selected to receive abdominal CT scans divided evenly between visits 2 and 3 (2002–2005). The study participants are roughly representative of the whole MESA cohort. We matched CT scans coded for the body composition ancillary study with measures of anthropometry by visit (n = 1851). Since thiazolidinediones have been shown to modify body fat composition and Cook’s Distance indicates influential outliers, we excluded participants with reported thiazolidinediones use (n = 20) or a Cook's Distance >0.025 (n = 30).[16]

Institutional Review Board approval was obtained at all MESA study sites (Columbia University, Johns Hopkins University, Northwestern University, UCLA, University of Minnesota, and Wake Forest University), and all participants provided written informed consent.

### Assessment of body fat composition

Abdominal CT scans were used to measure visceral and subcutaneous fat area by semi-automated segmentation of the body compartments and the Medical Image Processing, Analysis, and Visualization (MIPAV) algorithm from the National Institutes of Health.[17] Visceral and subcutaneous fat were defined, respectively, as the average of two measurements from CT slices at the L4/L5 vertebrae. The total area of the subcutaneous compartment was used for the measurement of subcutaneous fat to account for potential underestimation of subcutaneous fat area when measuring fat alone. The CT scans were read by three technicians with standardized training and were reviewed regularly for quality assurance. Inter and intra-rater reliabilities for total abdominal, subcutaneous, and visceral cavity areas were 0.99 for all measures. Repeat CT scans were available at visit 4 (2005–2007) for 650 participants and were read in the same way as the initial scans.

### Assessment of anthropometry

Anthropometry measures included height, weight, waist circumference, and hip circumference. Weight was measured using a balance beam scale to the nearest 0.5 lb. Height was measured using a vertical ruler to the nearest 0.5 cm. Waist circumference was measured at the minimum abdominal circumference, and hip circumference at the maximum girth at the level of the symphysis pubis, both to the nearest 0.1 cm.[18] All anthropometric components were measured twice by study staff using a standardized protocol and averaged. Derived variables included waist-to-height ratio, waist-to-hip ratio, and BMI.

### Covariates

Socio-economic and demographic factors such as sex, race/ethnicity, and age as well as health behaviors such as smoking, alcohol consumption, and leisure time physical activity were assessed by self-report at enrollment into the study. Medical conditions, treatment, and medication use were collected at baseline and updated at every visit. Medical conditions were based on study visit exams or by self-report with verification through examination of medication containers. Detailed documentation about variable collection can be found at: http://www.mesa-nhlbi.org/ex1forms.aspx#exam.

### Statistical Analysis

We used multivariable linear regression to determine relationships between anthropometry and body fat composition by sex and race/ethnicity. We performed separate analyses for visceral and subcutaneous fat. For the primary analyses we excluded values that were missing or imputed. In a cross-sectional sample from visits 2 and 3, we used F tests and likelihood ratio tests to investigate best fitting models focusing on interaction and non-linear terms. We matched anthropometry to CT derived measurements by visit. For example, we compared visceral fat at visit 2 with BMI for that participant at the same visit.

To address non-constant variance and a lack of symmetry (seen in standardized residuals), we used the natural log to transform visceral and subcutaneous fat values. We also centered each anthropometric variable to a value contained in the lower range of the distributions for both men and women. The centering was as follows: height—160cm, weight—50kg, BMI—20 kg/m^2^, waist circumference—100cm, hip circumference—100cm, waist to hip ratio—0.7, and waist to height ratio—0.4. We further investigated the functional form by including increasingly higher order polynomial terms until likelihood ratio tests indicated no significant improvement in the model. We formally tested for interaction by sex and race/ethnicity by including interaction terms in the regression models. Analyses were performed using Stata 11.

### Sensitivity Analyses

We conducted sensitivity analyses to determine if the relationship between BMI or waist circumference and visceral fat differed by various medical conditions, medication use, or individual health behaviors. Specifically, we investigated the influence of diabetes, thyroid conditions, hormone replacement therapy, menopausal status, and smoking behavior collected at the same visit as the CT scan. We also investigated whether these relationships differed by age and whether adjustment for leisure time exercise explained the results. Finally, we tested sensitivity to missing values by analyzing the association with visceral and subcutaneous fat using fully imputed values.

### Repeated Measures Analyses

There were 650 participants with repeated CT scans at the MESA study visit 4. In these participants we conducted our analyses again using average body fat composition measurements from the two CT scans in order to assess bias due to regression to the mean and regression dilution bias. We used values of anthropometry and body fat composition averaged from the first (visit 2 or 3) and second (visit 4) CT scans. We also used mixed linear models to conduct a repeated measures analysis between measures of anthropometry at all visits with measures of body fat composition from CT scans at two time points.

### Missing and Imputed Data

For our investigation of the relationship of anthropometric measures with visceral and subcutaneous fat all missing or imputed values were excluded. To assess the generalizability of our sample, we investigated mechanisms for missing data by determining if participants with missing or imputed visceral or subcutaneous fat measurements were systematically different from those with non-missing values with regard to mean anthropometric measurements. A summary of the imputation process is as follows: For some participants, the field of view was insufficient to measure the entire subcutaneous compartment (n = 543). Where the CT scan of the subcutaneous compartment was incomplete on one side of the body (n = 219), subcutaneous fat area was estimated as double the value of the symmetric other body half. Where half process estimation was not possible, estimating equations were used to calculate a predicted subcutaneous area from other variables such as sex, race/ethnicity, and anthropometric measures (n = 152). For a much smaller number of participants (n = 49), the CT scan of the visceral compartment was incomplete. For those with incomplete CT scans of the visceral compartment, where the visceral cavity was truncated on only one side and the truncation was less than 10 cm^2^ (n = 25), the missing visceral fat area was imputed by doubling the non-truncated area symmetric to the area of truncation, in a manner similar to that used for the half process imputation for subcutaneous fat.

## Results

The 1,851 participants included in the final analytic sample were 50% female, 41% Caucasian, 13% Asian, 21% African American, and 25% Hispanic. The mean age was 62 years at enrollment. In general, participants who had higher visceral fat also had significantly higher total abdominal fat mass and anthropometric measures (Table 1). They were also more likely to be Hispanic, older, have diabetes, and be post-menopausal. They were less likely to be female, have graduated from high school, or be taking hormone replacement therapy. Participants with higher levels of subcutaneous fat also had significantly higher total abdominal fat mass and anthropometric measures, except for height (Table 1). They were significantly more likely to be female or have a thyroid condition and less likely to be current alcohol drinkers.

**Table 1:** Characteristics (Mean (SD) or Percentile) of 1851 Adults^a^ Aged 45–84 in the MESA Body Composition Ancillary Study by Body Composition Quartile^b^.

**Ln Visceral fat** (n = 1851)	**Total**	**Quartile 1** (<4.58)	**Quartile 2** (4.58–4.93)	**Quartile 3** (4.93–5.27)	**Quartile 4** (5.26 ≤)	***P* for trend**
Ln visceral fat (cm^2^)	4.89 (0.012)	4.20	4.76	5.09	5.49	
Sex (% female)	50.2 (1.16)	67.5 (2.18)	52.4 (2.32)	46.7 (2.32)	34.4 (2.21)	<0.001
Race/Ethnicity						0.66
White (%)	40.6 (1.14)	40.0 (2.27)	30.6 (2.14)	42.1 (2.30)	50.9 (2.33)	
Chinese (%)	13.1 (0.78)	19.3 (1.84)	19.0 (1.82)	9.72 (1.38)	4.33 (0.95)	
Black (%)	20.9 (0.94)	27.9 (2.09)	25.9 (2.03)	16.6 (1.73)	13.0 (1.57)	
Hispanic (%)	25.4 (1.01)	13.9 (1.61)	24.6 (2.00)	31.5 (2.16)	31.8 (2.17)	
Age	61.9 (0.23)	60.5 (0.46)	61.3 (0.45)	62.3 (0.44)	63.7 (0.44)	<0.001
Total Gross Family Income (% ≥ $35,000)	54.8 (1.16)	58.9 (2.29)	51.7 (2.32)	55.3 (2.31)	53.5 (2.32)	0.22
Education (% completed high school)	82.4 (0.88)	87.2 (1.55)	81.6 (1.80)	81.4 (1.81)	79.4 (1.88)	0.003
Smoking (% current smoker)	13.0 (0.78)	13.6 (1.60)	12.1 (1.51)	11.7 (1.49)	14.7 (1.65)	0.69
Alcohol (% current drinker)	70.7 (1.18)	74.7 (2.29)	67.1 (2.50)	71.9 (2.31)	69.2 (2.32)	0.27
Cancer (% dx)	8.10 (0.63)	7.36 (1.22)	7.76 (1.24)	6.91 (1.18)	10.4 (1.42)	0.15
Thyroid Condition (% dx)	6.10 (0.56)	5.19 (1.03)	6.47 (1.14)	4.97 (1.01)	7.79 (1.25)	0.21
Diabetes (% dx)^c^	10.1 (0.70)	3.9 (0.90)	9.3 (1.35)	11.0 (1.46)	16.1 (1.71)	<0.001
Kidney Disease (% self-report)	2.11 (0.33)	1.95 (0.64)	2.37 (0.71)	1.73 (0.61)	2.38 (0.71)	0.35
Menopausal Status (% post menopausal)	90.9 (0.95)	87.5 (1.87)	91.8 (1.77)	89.8 (2.06)	97.5 (1.25)	0.002
Current HRT users (%)	34.6 (1.60)	39.0 (2.86)	36.4 (3.14)	28.6 (3.16)	31.4 (3.73)	0.023
Total abdominal fat mass (cm^2^)	413.8 (4.07)	282.5 (4.77)	387.3 (5.84)	456.6 (5.81)	585.7 (8.44)	<0.001
Height (cm)	166.3 (0.23)	164.2 (0.42)	166.0 (0.45)	166.5 (0.46)	168.6 (0.49)	<0.001
Weight (kg)	77.4 (0.38)	64.7 (0.56)	75.0 (0.62)	80.0 (0.59)	90.1 (0.71)	<0.001
Body Mass Index (kg/m^2^)	27.9 (0.12)	23.9 (0.16)	27.2 (0.19)	28.8 (0.18)	31.7 (0.22)	<0.001
Waist Circumference (cm)	97.6 (0.31)	85.1 (0.44)	95.1 (0.47)	100.5 (0.46)	109.8 (0.52)	<0.001
Hip Circumference (cm)	104.0 (0.24)	97.7 (0.38)	102.9 (0.44)	105.1 (0.40)	110.3 (0.51)	<0.001
Waist to Hip Ratio	0.94 (0.002)	0.87 (0.003)	0.92 (0.003)	0.96 (0.003)	1.00 (0.002)	<0.001
Waist to Height Ratio	0.59 (0.002)	0.52 (0.003)	0.57 (0.003)	0.61 (0.003)	0.65 (0.004)	<0.001
**Ln Subcutaneous fat** (n = 1754)						
	**Total**	**Quartile 1** (<5.14)	**Quartile 2** (5.14–5.46)	**Quartile 3** (5.46–5.74)	**Quartile 4** (5.74 ≤)	***P* for trend**
Average ln subcutaneous fat (cm^2^)	5.44 (0.012)	4.86	5.32	5.59	6.01	
Sex (% female)	47.1 (1.28)	28.5 (2.43)	39.7 (2.63)	53.4 (2.68)	71.8 (2.42)	<0.001
Race/Ethnicity						0.002
White (%)	40.8 (1.26)	40.1 (2.63)	41.4 (2.64)	29.7 (2.62)	41.7 (2.66)	
Chinese (%)	14.2 (0.89)	30.8 (2.48)	20.1 (2.15)	7.8 (1.44)	2.9 (0.90)	
Black (%)	21.4 (1.05)	13.5 (1.84)	15.8 (1.96)	24.4 (2.31)	32.6 (2.52)	
Hispanic (%)	23.6 (1.09)	15.6 (1.95)	22.7 (2.25)	28.2 (2.41)	21.9 (2.22)	
Age	61.9 (0.25)	52.1 (0.55)	62.2 (0.55)	62.2 (0.50)	60.5 (0.47)	0.065
Total Gross Family Income (% ≥ $35,000)	54.4 (1.27)	57.3 (2.66)	54.6 (2.67)	54.9 (2.67)	53.69 (2.68)	0.36
Education (% completed high school)	82.7 (0.97)	84.7 (1.94)	81.3 (2.09)	83.6 (1.99)	83.0 (2.02)	0.76
Smoking (% current smoker)	13.2 (0.86)	13.5 (1.84)	11.2 (1.69)	12.1 (1.75)	14.7 (1.90)	0.59
Alcohol (% current drinker)	70.5 (1.30)	71.4 (2.73)	77.1 (2.51)	72.9 (2.67)	60.8 (2.93)	0.003
Cancer (% dx cancer)	8.16 (0.80)	6.92 (1.36)	7.47 (1.41)	8.33 (1.48)	9.80 (1.60)	0.15
Thyroid Condition (% dx)	5.61 (0.59)	2.88 (0.90)	4.89 (1.16)	6.03 (1.28)	8.07 (1.46)	0.002
Diabetes (% dx)	10.0 (0.77)	6.63 (1.34)	8.93 (1.53)	9.25 (1.56)	12.4 (1.77)	0.012
Kidney Disease (% dx)	2.29 (0.38)	1.73 (0.70)	3.16 (0.94)	1.44 (0.64)	2.59 (0.85)	0.41
Menopausal Status (% post menopausal)	90.8 (1.07)	92.9 (2.59)	91.3 (2.41)	87.1 (2.46)	92.4 (1.69)	0.33
Current HRT users (%)	35.5 (1.82)	44.7 (5.16)	34.1 (4.14)	37.6 (3.69)	35.6 (3.05)	0.29
Total abdominal fat mass (cm^2^)	406.0 (4.32)	241.5 (3.97)	352.1 (3.98)	425.8 (3.97)	604.6 (7.06)	<0.001
Height (cm)	466.7 (0.26)	167.2 (0.49)	167.1 (0.54)	166.1 (0.56)	165.2 (0.53)	0.002
Weight (kg)	76.6 (0.42)	64.2 (0.55)	71.8 (0.68)	75.8 (0.72)	88.16 (0.83)	<0.001
Body Mass Index (kg/m^2^)	27.5 (0.12)	22.8 (0.13)	25.6 (0.14)	27.3 (0.15)	32.2 (0.23)	<0.001
Waist Circumference (cm)	96.4 (0.34)	84.0 (0.42)	91.7 (0.43)	96.2 (0.47)	108.1 (0.67)	<0.001
Hip Circumference (cm)	103.0 (0.25)	93.9 (0.27)	98.5 (0.28)	102.7 (0.28)	113.3 (0.44)	<0.001
Waist to Hip Ratio	0.93 (0.002)	0.90 (0.004)	0.93 (0.004)	0.94 (0.004)	0.95 (0.004)	<0.001
Waist to Height Ratio	0.58 (0.002)	0.50 (0.002)	0.55 (0.002)	0.58 (0.003)	0.66 (0.004)	<0.001

a. Participants on Thiazolidinediones and observations with Cook's Distance >0.025 excluded.

b. Quartile cutoffs are equivalent to 97.7, 138.2, 193.6 cm^2^ visceral fat170.7, 235.1, 311.1 cm^2^ subcutaneous fat on the original scale

c. Diabetes diagnosed as ≥126 mg/dl fasting glucose

**Fig 1:**
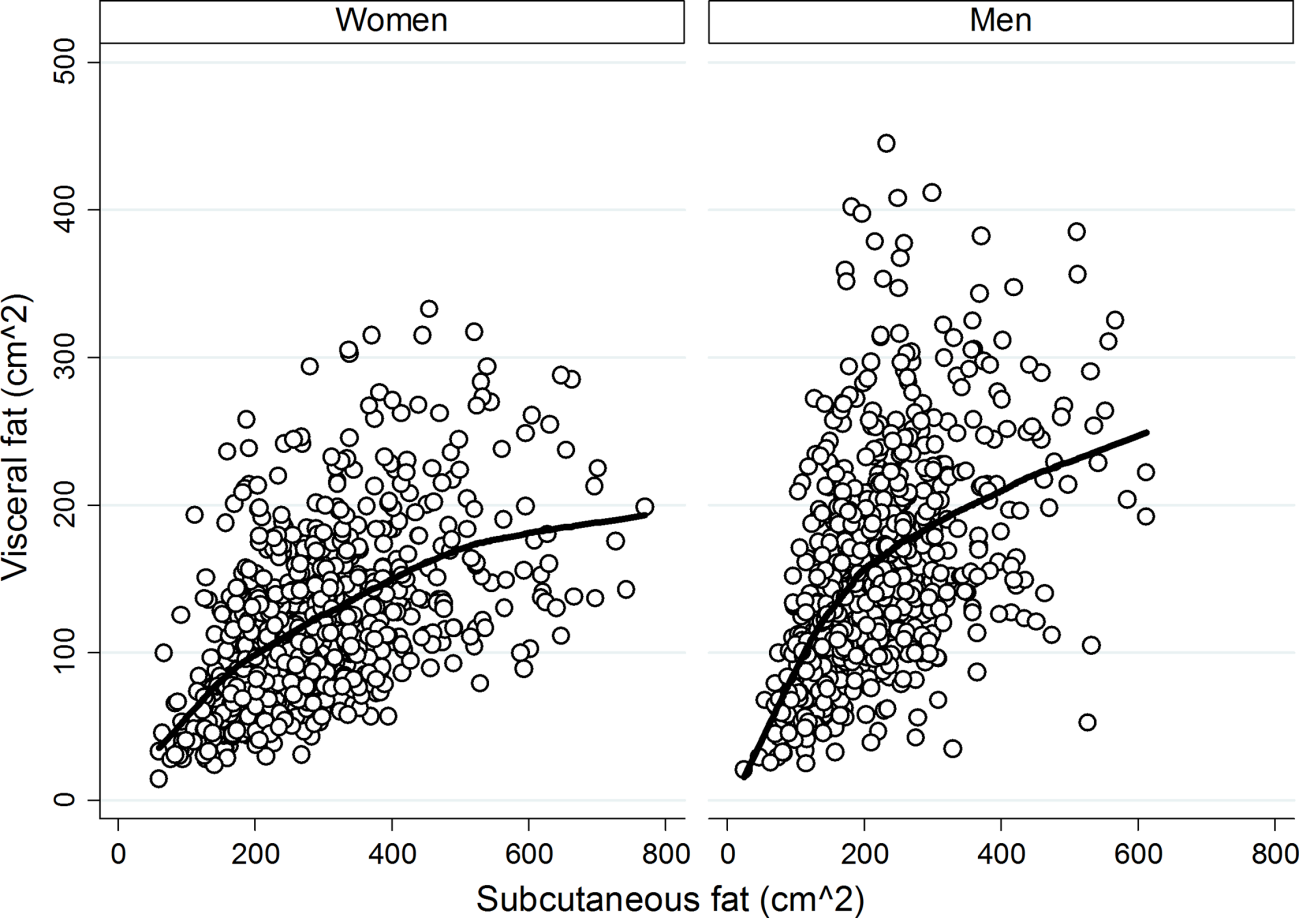
Lowess of Visceral Fat and Subcutaneous Fat by Sex in 1851 Adults in the MESA Body Composition ancillary Study.

In general, individuals with higher subcutaneous fat also had higher visceral fat (Fig 1). Women had more subcutaneous, but less visceral fat than men (Ln visceral fat intercept for men is 0.43cm^2^ (95% confidence interval = 0.39–0.47) higher than for women).

**Fig 2:**
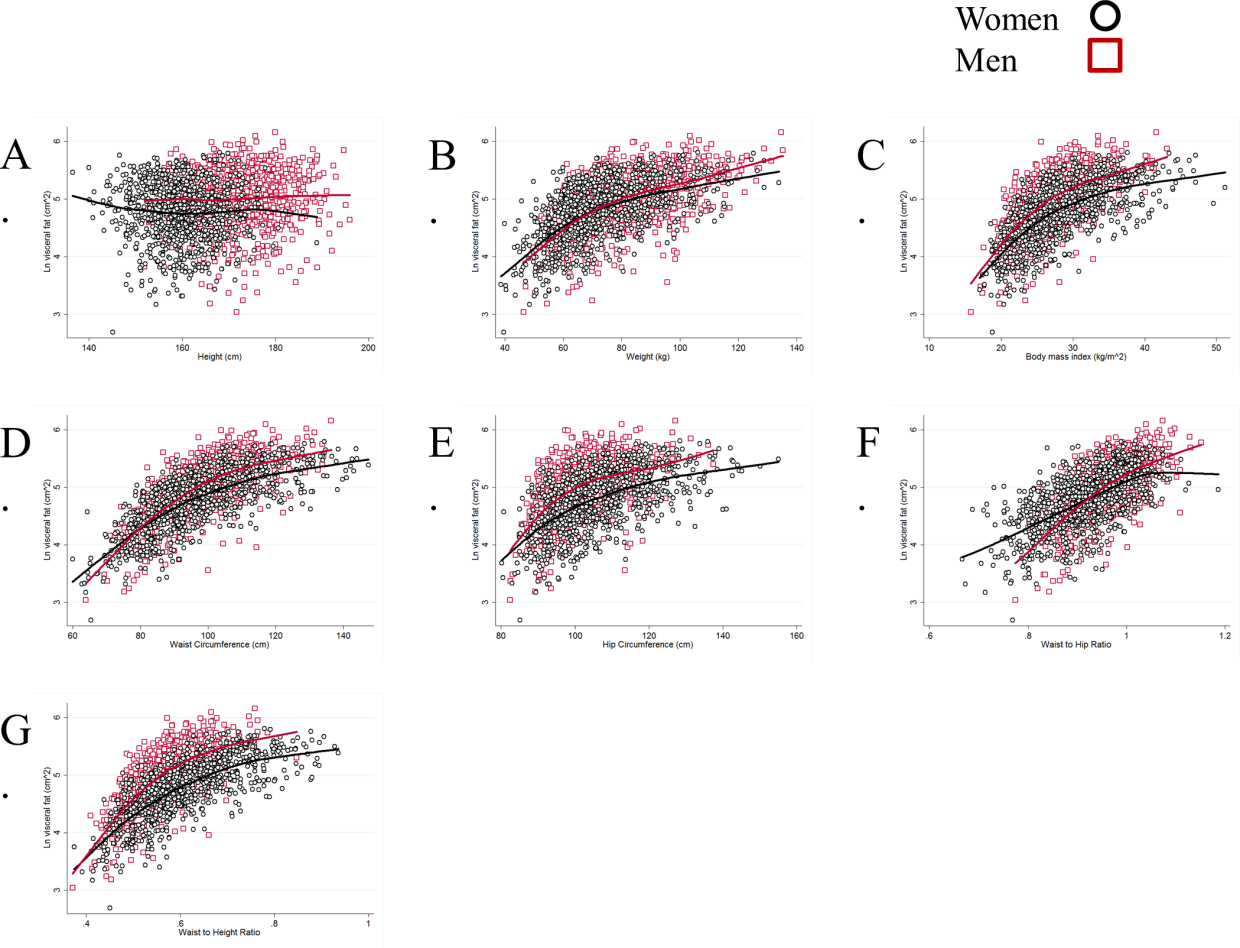
Lowess of ln Visceral Fat and Anthropometry by Sex in1851 Adults in the MESA Body Composition Ancillary Study.

All anthropometric indices, except height, were significantly associated with visceral fat at the p<0.05 level (Table 2 and Fig 2). These associations included a significant quadratic non-linear component for all anthropometric indices except for height. The slopes for weight, waist circumference, waist to hip ratio, and waist to height ratio and visceral fat were significantly steeper for men than for women (p<0.05) (Table 2). That is, across the range of these anthropometric measures the rise in visceral fat is faster for men than for women. Estimates for BMI and hip circumference did not significantly differ for men and women.

**Table 2:** Association between body fat composition measures and anthropometry by sex in MESA.

X		Intercept	+	Linear	+	Quadratic	P-value for sex difference
**Ln Visceral fat (cm** ^**2**^ **)**							
**Height** (cm)	*Female*	4.75		-0.0031		0.0002	0.82
	*Male*	4.99		-0.0020		0.0002	
**Weight** (kg)	*Female*	4.22		0.033		-0.003	0.028
	*Male*	4.16		0.036		-0.003	
**BMI** (kg/m^2^)	*Female*	4.07		0.12		-0.003	0.37
	*Male*	4.29		0.12		-0.003	
**Waist** (cm)	*Female*	4.94		0.024		-0.0002	<0.001
	*Male*	5.11		0.031		-0.0002	
**Hip** (cm)	*Female*	4.66		0.034		-0.005	0.63
	*Male*	4.97		0.033		-0.005	
**Waist to Hip**	*Female*	3.74		6.06		-5.22	<0.001
	*Male*	3.25		8.20		-5.22	
**Waist to Height**	*Female*	3.54		8.53		-10.3	0.050
	*Male*	3.83		8.97		-10.3	
**Ln Subcutaneous fat (cm** ^**2**^ **)**							
**Height** (cm)	*Female*	5.60		0.007		-0.00005	0.129
	*Male*	5.13		0.015		-0.00009	
**Weight** (kg)	*Female*	5.08		0.032		-0.0002	0.001
	*Male*	4.42		0.035		-0.0002	
**BMI** (kg/m^2^)	*Female*	5.00		0.11		-0.002	<0.001
	*Male*	4.58		0.12		-0.002	
**Waist** (cm)	*Female*	5.78		0.023		-0.0002	<0.001
	*Male*	5.45		0.033		-0.0002	
**Hip** (cm)	*Female*	5.51		0.040		-0.0004	<0.001
	*Male*	5.27		0.046		-0.0004	
**Waist to Hip**	*Female*	5.10		2.71		-1.42	<0.001
	*Male*	4.22		4.70		-1.42	
**Waist to Height**	*Female*	4.77		5.49		-4.63	<0.001
	*Male*	4.30		7.20		-4.63	

Regression equation for body fat composition by anthropometry and sex: Ln body fat composition = β0_1_ + β0_2_(sex) + β1(X) + β2(X^2^) + β3(sex*X). Intercept = β0_1_ + β0_2_, Linear = β1 **+** β3, Quadratic = β2, P-value for difference by sex = p-value for β3. Centering: height—160cm, weight—50kg, BMI—20 kg/m^2^, waist—100cm, hip—100cm, waist to hip—0.7, waist to height—0.4.

In general, the visceral fat relationships that displayed the most heterogeneity by sex also displayed the most heterogeneity by race/ethnicity (Table 3). This heterogeneity was predominantly characterized by the Hispanic group having a significantly shallower visceral fat slope than that for the Caucasian group for height, weight, waist circumference, hip circumference, and waist to hip ratio (S1 Table. Race/ethnicity by sex interactions for visceral fat and anthropometry). Sex by race/ethnicity interactions for subcutaneous fat also show that when heterogeneity is present, it includes both race/ethnicity and sex (S2 Table. Race/ethnicity by sex interactions for subcutaneous fat and anthropometry).

**Table 3:** Association Between Body Fat Composition Measures and Anthropometry by Race/Ethnicity in MESA.

X		Intercept	+	Linear	+	Quadratic	*P*-value for race difference
**Ln Visceral fat (cm** ^**2**^ **)**							
**Height** (cm)	*White*	4.76		0.008		-	<0.001
	*Asian*	6.65		-0.005		-	
	*Black*	6.73		-0.005		-	
	*Hispanic*	7.19		-0.007		-	
**Weight** (kg)	*White*	4.08		0.041		-0.0003	<0.001
	*Asian*	4.60		0.035		-0.0003	
	*Black*	4.11		0.037		-0.0003	
	*Hispanic*	4.71		0.034		-0.0003	
**BMI** (kg/m^2^)	*White*	4.08		0.132		-0.003	0.14
	*Asian*	4.16		0.129		-0.003	
	*Black*	3.94		0.126		-0.003	
	*Hispanic*	4.30		0.122		-0.003	
**Waist** (cm)	*White*	5.00		0.029		-0.0004	<0.001
	*Asian*	5.26		0.027		-0.0004	
	*Black*	5.14		0.025		-0.0004	
	*Hispanic*	5.39		0.025		-0.0004	
**Hip** (cm)	*White*	4.68		0.040		-0.0006	0.0063
	*Asian*	4.93		0.038		-0.0006	
	*Black*	4.79		0.036		-0.0006	
	*Hispanic*	5.56		0.032		-0.0006	
**Waist to Hip**	*White*	3.74		5.336		-1.21	0.0011
	*Asian*	4.08		4.749		-1.21	
	*Black*	4.73		4.122		-1.21	
	*Hispanic*	4.18		4.837		-1.21	
**Waist to Height**	*White*	3.56		8.90		-10.50	0.67
	*Asian*	3.48		8.88		-10.50	
	*Black*	3.52		8.61		-10.50	
	*Hispanic*	3.55		8.76		-10.50	
**Ln Subcutaneous fat (cm** ^**2**^ **)**							
**Height** (cm)	*White*	5.56		0.008		0.00008	0.35
	*Asian*	6.15		0.003		0.00008	
	*Black*	6.42		0.004		0.00008	
	*Hispanic*	6.31		0.004		0.00008	
**Weight** (kg)	*White*	5.02		0.032		-0.0001	0.61
	*Asian*	5.13		0.031		-0.0001	
	*Black*	4.95		0.033		-0.0001	
	*Hispanic*	5.12		0.032		-0.0001	
**BMI** (kg/m^2^)	*White*	4.96		0.117		-0.002	0.073
	*Asian*	5.08		0.108		-0.002	
	*Black*	4.93	0.118	-0.002	
	*Hispanic*	5.13		0.109		-0.002	
**Waist** (cm)	*White*	5.77		0.026		-0.0002	0.95
	*Asian*	5.63		0.027		-0.0002	
	*Black*	5.91		0.026		-0.0002	
	*Hispanic*	5.76		0.026		-0.0002	
**Hip** (cm)	*White*	5.47		0.044		-0.0005	0.068
	*Asian*	5.73		0.041		-0.0005	
	*Black*	5.32		0.046		-0.0005	
	*Hispanic*	5.94		0.04		-0.0005	
**Waist to Hip**	*White*	5.03		2.415		1.31	0.29
	*Asian*	5.14		1.977		1.31	
	*Black*	5.61		1.965		1.31	
	*Hispanic*	5.61		1.790		1.31	
**Waist to Height**	*White*	4.61		7.05		-7.24	0.49
	*Asian*	4.67		6.58		-7.24	
	*Black*	4.73		7.04		-7.24	
	*Hispanic*	4.66		6.83		-7.24	

Regression equation for body fat composition by anthropometry and race/ethnicity: Ln body fat composition = β0_1_ + β0_2_(race) + β1(X) + β2(X^2^) + β3(race*X). Intercept = β0_1_ + β0_2_, Linear = β1 **+** β3, Quadratic = β2, P-value for difference by race = *P*-value for overall F-test. Centering: height—160cm, weight—50kg, BMI—20 kg/m^2^, waist—100cm, hip—100cm, waist to hip—0.7, waist to height—0.4.

All anthropometric indices were significantly associated with subcutaneous fat, with a significantly steeper slope for men than for women. The overall F-tests for subcutaneous fat indicated no significant differences by race/ethnicity (Table 2).

### Sensitivity Analyses, Average Values, and Repeated Measures

Table 4 shows the results of the subgroup analyses by type 2 diabetes, thyroid conditions, hormone replacement therapy, menopausal status, and smoking behavior. None of the subgroup estimates were significantly different from the full group estimate. Additionally, we observed no significant heterogeneity by age (data not shown). Adjusting for leisure time exercise did not explain our results. Estimates using averaged values of anthropometry and body fat composition and repeated measures analysis were similar to those using only one measurement (Table 4), probably due to the majority of the variance coming from between subject differences even using measurements from two time points for a small subset of participants. Including imputed values of visceral or subcutaneous fat also produced similar results (data not shown).

**Table 4:** Sensitivity and repeated measures analysis.

		BMI			Waist Circumference		
Estimates		Slope for Women	Slope for Men	P-value for Sex Difference	Slope for Women	Slope for Men	P-value for Sex Difference
**Single measure vs. repeated measures**							
**Reported Estimates**	0.12 (0.11, 0.13)	0.12 (0.11, 0.14)	0.37	0.025 (0.023, 0.026)	0.030 (0.026, 0.033)	<0.001
**Average**		0.12 (0.10, 0.14)	0.12 (0.11, 0.15)	0.99	0.024 (0.021, 0.027)	0.027 (0.023, 0.34)	0.18
**Repeated Measures**	0.12 (0.11, 0.13)	0.13 (0.11, 0.14)	0.095	0.022 (0.021, 0.024)	0.030 (0.026, 0.034)	<0.001
**Subgroup Analysis**							
**Full Group**		0.12 (0.11, 0.13)	0.14 (0.12, 0.15)	0.36	0.024 (0.023, 0.025)	0.031 (0.029, 0.033)	<0.001
**Cancer**	N	0.12 (0.10, 0.13)	0.13 (0.11, 0.15)	0.27	0.024 (0.022, 0.025)	0.031 (0.029, 0.033)	<0.001
	Y	0.13 (0.10, 0.17)	0.13 (0.07, 0.19)	0.71	0.025 (0.020, 0.029)	0.028 (0.020, 0.037)	0.37
**Thyroid**	N	0.12 (0.11, 0.13)	0.13 (0.12, 0.15)	0.32	0.024 (0.023, 0.026)	0.031 (0.029, 0.033)	<0.001
	Y	0.11 (0.08, 0.16)	0.14 (0.12, 0.23)	0.49	0.022 (0.017, 0.027)	0.043 (0.018, 0.068)	0.046
**Diabetes**	N	0.12 (0.08, 0.15)	0.16 (0.12, 0.19)	0.79	0.017 (0.013, 0.021)	0.030 (0.025, 0.034)	<0.001
	Y	0.09 (0.05, 0.14)	0.10 (0.07, 0.18)	0.58	0.021 (0.016, 0.027)	0.030 (0.022, 0.037)	0.04
**Smoking**	N	0.12 (0.11, 0.13)	0.13 (0.11, 0.15)	0.66	0.024 (0.022, 0.026)	0.031 (0.029, 0.033)	<0.001
	Y	0.12 (0.09, 0.15)	0.13 (0.10, 0.16)	0.27	0.024 (0.020, 0.028)	0.033 (0.028, 0.034)	0.005
**Menopausal** ^b^	N	0.09 (0.05, 0.14)		0.21	0.021 (0.016, 0.027)		0.002
	Y	0.12 (0.11, 0.13)		0.53	0.024 (0.023, 0.026)		0.002
**HRT Use** ^a^ ^,^ ^b^	N	0.11 (0.10, 0.13)		0.20	0.025 (0.023, 0.027)		<0.001
	Y	0.12 (0.10, 0.14)		0.98	0.023 (0.021, 0.025)		<0.001

All models include ln visceral fat = sex + BMI + BMI^2^ + BMI*sex OR ln visceral fat = sex + waist + waist^2^ + waist*sex. For reported estimates n = 1851, for full group estimates n = 1876, for average and repeated measures estimates n = 507. No statistically significant difference between subgroups or between any subgroup and full group estimate.

a. HRT = Hormone replacement therapy

b. All t-tests for sex differences for menopausal status and HRT are compared to men's full group estimates.

### Missing and Imputed Data

Participants who had missing values for body fat composition from CT scans had significantly higher anthropometric measure values than those with non-missing values (S3 Table. Anthropometry by visceral fat missing status in the MESA body composition ancillary study). Participants with missing subcutaneous fat measurements filled with half process of regression imputation had values for anthropometric measures in between those with completely missing and fully measured subcutaneous fat measurements (S4 Table. Anthropometry by subcutaneous fat (cm^2^) missing status in the MESA body composition ancillary study). The most common reason for missing body fat composition data was insufficient CT scan field of view to completely capture the specified body compartment.

## Discussion

We found that the relationship between anthropometric indices and visceral fat included significant non-linearity and some heterogeneity by sex and race/ethnicity. Visceral fat heterogeneity was most evident when weight and waist circumference were evaluated. The relationships with subcutaneous fat exhibited similar non-linearity and overall differences by sex, but not by race/ethnicity. These results were supported by sensitivity and repeated measures analysis.

Our finding of an interaction by sex for waist circumference, but not for BMI in predicting visceral fat supports current practice. While the controversy over the need for separate values of BMI continues, most researchers use the same values for both sexes and the underlying reasons for the difference in the relationship for waist circumference compared to BMI have yet to be fully explained. Particularly given the theory that waist circumference is more closely associated to visceral fat than other measures of anthropometry, it is confusing that there is statistically significant interaction for the relationship for waist circumference, but not for BMI. More specifically, the null results for interaction by sex for BMI, while consistent with common practice, remain inexplicable. While the consistently higher level of visceral fat at the same level of BMI for men compared to women supports the use of different cutoff values, the null interaction suggests that adjusting for sex in models that use BMI as a predictor is appropriate. One possibility for the null BMI interaction by sex is that, given the very different distributions in height between men and women, the inclusion of height in the calculation of BMI acts as a proxy for sex differences in stature and therefore accounts for the differences in the relationship with visceral fat.[19] In order to better understand the mechanisms underlying sex differences in CVD risk and the role that adiposity plays in those differences, we should determine why visceral fat differences by sex exist for waist circumference, but not for BMI.

While our results support current practice and our general conclusion that the relationship between anthropometry and adiposity may differ by sex and race/ethnicity is consistent with other studies, some studies found interaction for different anthropometric measures or among different groups.[12, 14, 20] In contrast to our findings for visceral fat, Camhi et al. found an interaction by sex for BMI, but not for waist circumference. In contrast to our findings for subcutaneous fat, they did not find anthropometry by sex interactions. The differences in findings might be explained by differences in study populations and analytic approaches. Specifically, our participants were older on average, visceral and subcutaneous fat values were transformed, and non-linearity in the relationship of anthropometric measures with adiposity was accounted for. Perhaps more importantly, the participants of the Pennington Center Longitudinal Study may have participated in interventions specifically targeted at weight loss, nutrition, or metabolic health.[20]

Two other studies did not formally test for interaction by sex or for the inclusion of non-linear terms in their models.[12, 14] We therefore cannot assess how differences by sex or non-linearity in these studies might compare to our results. Both studies found interaction by race, but the specifics of this interaction differ from our results.[12, 14] These inconsistencies might be a result of differences in the ethnic composition of the Hispanic and Asian groups among the three studies.

Consistent with our results, Schreiner et al. showed that appropriate modeling strategies using traditional anthropometric variables as markers of visceral fat mass may be different by sex in the Atherosclerosis Risk In Communities MRI study.[21] In particular, they showed that some of these relationships may be non-linear and that the slopes were steeper for men in the lower part of the anthropometric distributions than for women. Oka et al. had similar results in a large study of Japanese men and women.[22]

Our study has a number of limitations. First, despite the large sample size and oversampling of minority groups, there is limited power to assess sex by race/ethnicity interaction. Further, due to the homogeneity of the Asian (primarily Chinese Americans) population in MESA, the generalizability of results from MESA may be limited. While MESA participants who self-identified as Hispanic are more diverse, they are predominantly of Mexican, Dominican, and Puerto Rican descent. Second, this study only makes use of the average of two CT slices at the L4/L5 vertebrae. While it has been suggested that this slice may not be the best approximation of the total volume from the visceral compartment, [23] and that the best location may differ by sex and race,[13, 23] we chose the L4/L5 slice for the highest level of comparability across studies. The third limitation of this study is the amount of differential missing data at the high end of the anthropometry and body fat composition ranges. While the missing data limit inference at the highest levels of anthropometry, there is little need for evidence about the relative level of visceral or subcutaneous fat for a participant with a BMI > 40kg/m^2^ or a waist circumference > 120cm, as they are already considered high risk. Finally, since the body composition ancillary study is primarily cross-sectional, our ability to assess the longitudinal relationship between anthropometry and body fat composition was limited.

Our study also has a number of strengths. To our knowledge this is the most comprehensive investigation of the association of anthropometry with visceral and subcutaneous fat, accounting for non-linearity as well as interaction by sex and race/ethnicity. The comprehensive nature of our study is due in large part to the large sample size and diversity of the MESA body composition ancillary study. The large and diverse study population allows for investigation of differences by sex and race/ethnicity, and oversampling for minority groups in MESA allows sufficient power to determine relationships within each group. The CT scans from the body composition ancillary study enable comparison between anthropometry and visceral fat, the gold standard measurement of adiposity. Finally, the richness of data available in the MESA study makes subgroup and sensitivity analysis possible.

This study supports existing evidence that the relationship between anthropometry and body fat composition is non-linear and differs by race/ethnicity and sex. By improving the approximation of visceral fat by anthropometric measures, it may be possible to increase the precision of estimates derived from models that include anthropometric measures as predictors, outcomes, or confounders. Given the ubiquitous use of anthropometry as proxy for body fat composition and the increasing concerns over obesity, such increases in precision might make subtle but important differences in research. Taking these aspects into consideration may lead to further discovery of what causes variation in body fat composition and how adiposity affects CVD risk and other health outcomes.

## Supporting Information

S1 Table Race/ethnicity by sex interactions for visceral fat and anthropometry. (PDF)

S2 Table Race/ethnicity by sex interactions for subcutaneous fat and anthropometry. (PDF)

S3 Table Anthropometry by visceral fat missing status in the MESA body composition ancillary study. (PDF)

S4 Table Anthropometry by subcutaneous fat (cm^2^) missing status in the MESA body composition ancillary study. (PDF)

## Abbreviations

BMI: Body mass index
CVD: Cardiovascular disease
CT: Computed tomography
MIPAV: Medical Image Processing, Analysis, and Visualization
MESA: Multi-Ethnic Study of Atherosclerosis
