# Supplementary material for: The Sex and Race Specific Relationship between Anthropometry and Body Fat Composition Determined from Computed Tomography: Evidence from the Multi-Ethnic Study of Atherosclerosis
Source: PLoS One. 2015 Oct 8;10(10):e0139559. doi: 10.1371/journal.pone.0139559 (PMC4598154; doi:10.1371/journal.pone.0139559)
Supplement: S1 Table — (PDF) [file pone.0139559.s001.pdf]

S1 Table. Race/ethnicity by sex interactions for visceral fat and anthropometry

			Women			Men	
**Measure**	**Race**	**White**	**Asian**	**Black**	**White**	**Asian**	**Black**
**Height** (cm)	Asian						
	Black						
	Hispanic	***	*	*	***		*
**Weight** (kg)	Asian						
	Black				*		
	Hispanic	***		*	***		*
**BMI** (kg/m^2)	Asian						
	Black						
	Hispanic	*			*		
**Waist** (cm)	Asian	**			*		
	Black	**	***		***	***	
	Hispanic	***	***		***	***	
**Hip** (cm)	Asian						
	Black				*		
	Hispanic	***			***		
**Waist to Hip**	Asian						
	Black	*			**		
	Hispanic						
**Waist to Height**	Asian						
	Black				*		
	Hispanic						

*<0.05 **<0.01 ***<0.001
