# Supplementary material for: The Sex and Race Specific Relationship between Anthropometry and Body Fat Composition Determined from Computed Tomography: Evidence from the Multi-Ethnic Study of Atherosclerosis
Source: PLoS One. 2015 Oct 8;10(10):e0139559. doi: 10.1371/journal.pone.0139559 (PMC4598154; doi:10.1371/journal.pone.0139559)
Supplement: S2 Table — (PDF) [file pone.0139559.s002.pdf]

S2 Table. Race/ethnicity by sex interactions for subcutaneous fat and anthropometry

		Women			Men		
**Measure**	**Race**	**White**	**Asian**	**Black**	**White**	**Asian**	**Black**
**Height** (cm)	Asian						
	Black						
	Hispanic						
**Weight** (kg)	Asian						
	Black						
	Hispanic						
**BMI** (kg/m^2)	Asian						
	Black						
	Hispanic	*		**	*		**
**Waist** (cm)	Asian						
	Black						
	Hispanic						
**Hip** (cm)	Asian						
	Black						
	Hispanic	*		**			*
**Waist to Hip**	Asian						
	Black						
	Hispanic						
**Waist to Height**	Asian						
	Black						
	Hispanic						

*<0.05 **<0.01 ***<0.001
