# Supplementary material for: The Sex and Race Specific Relationship between Anthropometry and Body Fat Composition Determined from Computed Tomography: Evidence from the Multi-Ethnic Study of Atherosclerosis
Source: PLoS One. 2015 Oct 8;10(10):e0139559. doi: 10.1371/journal.pone.0139559 (PMC4598154; doi:10.1371/journal.pone.0139559)
Supplement: S3 Table — (PDF) [file pone.0139559.s003.pdf]

**S3 Table. Anthropometry by visceral fat missing status in the MESA body composition ancillary study**

| Variable | Non-Missing Mean (SD) | Missing Visceral Fat Mean (SD) | Imputed Visceral Fat Mean (SD) | P-value: Non-Missing vs. Missing |
|---|---|---|---|---|
| N | 1898 | 24 | 25 | |
| Visceral fat ($cm^2$) | 149.6 (1.61) | NA | 258.8 (8.13) | |
| Age (years) | 62.0 (0.22) | 64.8 (1.91) | 64.3 (2.13) | <0.001 |
| Female (%) | 50.1 (1.15) | 16.7 (7.78) | 32.0 (9.52) | <0.001 |
| | | | | |
| Height (cm) | 166.3 (0.23) | 170.7 (1.51) | 170.7 (2.82) | <0.001 |
| Weight (kg) | 77.6 (0.38) | 101.2 (4.02) | 97.3 (2.79) | <0.001 |
| BMI ($kg/m^2$) | 28.0 (0.12) | 34.6 (1.14) | 33.6 (0.98) | <0.001 |
| Waist circumference (cm) | 97.8 (0.32) | 118.5 (2.70) | 113.6 (1.92) | <0.001 |
| Hip Circumference (cm) | 104.1 (0.25) | 115.4 (2.12) | 114.3 (2.06) | <0.001 |
| Waist to Hip Ratio | 0.94 (0.002) | 1.03 (0.013) | 1.00 (0.009) | <0.001 |
| Waist to Height Ratio | 0.59 (0.002) | 0.69 (0.015) | 0.67 (0.019) | <0.001 |
