# Supplementary material for: The Sex and Race Specific Relationship between Anthropometry and Body Fat Composition Determined from Computed Tomography: Evidence from the Multi-Ethnic Study of Atherosclerosis
Source: PLoS One. 2015 Oct 8;10(10):e0139559. doi: 10.1371/journal.pone.0139559 (PMC4598154; doi:10.1371/journal.pone.0139559)
Supplement: S4 Table — (PDF) [file pone.0139559.s004.pdf]

S4 Table. Anthropometry by subcutaneous fat (cm^2) missing status in the MESA body composition ancillary study

Variable	Directly Measured Mean (SD)	Completely Missing Mean (SD)	Filled with half process Mean (SD)	Filled with regression equations Mean (SD)	P-value: Measured vs. Missing	P-value: Measured vs. Half process	P-value: Measured vs. Regression
N	1,404	172	219	152			
Age (years)	61.80 (0.26)	63.55 (0.77)	62.41 (0.28)	62.41 (0.78)	0.030	0.40	0.45
Female (%)	48.29 (1.33)	32.56 (3.58)	57.53 (3.35)	67.76 (3.80)	<0.001	0.010	<0.001
Height (cm)	166.37 (0.26)	169.51 (0.78)	165.27 (0.69)	164.92 (0.74)	<0.001	0.14	0.064
Weight (kg)	75.01 (0.42)	94.55 (1.26)	78.60 (0.98)	88.11 (1.22)	<0.001	0.001	<0.001
BMI (kg/m^2)	26.99 (0.12)	32.98 (0.42)	28.72 (0.30)	32.43 (0.42)	<0.001	<0.001	<0.001
Waist circumference (cm)	95.03 (0.35)	112.31 (1.00)	100.10 (0.77)	109.60 (1.00)	<0.001	<0.001	<0.001
Hip Circumference (cm)	102.11 (0.25)	113.28 (0.92)	105.32 (0.62)	114.06 (0.99)	<0.001	<0.001	<0.001
Waist to Hip Ratio	0.93 (0.002)	0.99 (0.005)	0.95 (0.005)	0.96 (0.006)	<0.001	<0.001	<0.001
Waist to Height Ratio	0.57 (0.002)	0.67 (0.007)	0.61 (0.005)	0.67 (0.007)	<0.001	<0.001	<0.001
